# Comparative Transcriptome Analyses Provide New Insights into the Evolution of Divergent Thermal Resistance in Two Eel Gobies

**DOI:** 10.3390/cimb46010012

**Published:** 2023-12-25

**Authors:** Jing Liu, Tianwei Liu, Yantao Liu, Yuzhen Wang, Liqin Liu, Li Gong, Bingjian Liu, Zhenming Lü

**Affiliations:** 1 National Engineering Laboratory of Marine Germplasm Resources Exploration and Utilization, College of Marine Sciences and Technology, Zhejiang Ocean University, Zhoushan 316022, China; 2022157@zjou.edu.cn (J.L.); liutianwei@zjou.edu.cn (T.L.); liuyantao@zjou.edu.cn (Y.L.); liulq@zjou.edu.cn (L.L.); gongli@zjou.edu.cn (L.G.); liubingjian@zjou.edu.cn (B.L.); 2 National Engineering Research Center for Facilitated Marine Aquaculture, Zhejiang Ocean University, Zhoushan 316022, China; wangyuzhen@zjou.edu.cn

**Keywords:** comparative transcriptomics, thermal adaptation, genetic mechanisms, eel goby

## Abstract

Adaptation to thermal conditions in tidal mudflats always involves tolerating frequent fluctuations and often extreme environmental temperatures. Regulation of gene expression plays a fundamental role in the evolution of these thermal adaptations. To identify the key gene regulatory networks associated with the thermal adaptation, we investigated the capability of cold tolerance, as well as the transcriptomic changes under cold stress in two mudflat inhabitants (*Odontamblyopus lacepedii* and *O. rebecca*) with contrasting latitude affinity. Our results revealed a remarkable divergent capacity of cold tolerance (CT_min_: 0.61 °C vs. 9.57 °C) between the two gobies. Analysis of transcriptomic changes under cold stress unveiled 193 differentially expressed genes exhibiting similar expression profiles across all tissues and species, including several classic metabolic and circadian rhythm molecules such as *ACOD* and *CIART* that may represent the core cold response machinery in eel gobies. Meanwhile, some genes show a unique expression spectrum in the more cold-tolerant *O. lacepedii* suggesting their roles in the enhanced cold tolerance and hence the extreme thermal adaptations. In addition, a weighted gene co-expression network analysis (WGCNA) revealed a subset of metabolic hub genes including *MYH11* and *LIPT2* showing distinct down-regulation in *O. lacepedii* when exposed to cold stress which highlights the role of reduced energy consumption in the enhanced cold tolerance of eel gobies. These findings not only provide new insights into how mudflat teleosts could cope with cold stress and their potential evolutionary strategies for adapting to their thermal environment, but also have important implications for sound management and conservation of their fishery resources in a scenario of global climate warming in the marine realm.

## 1. Introduction

Thermal adaptation is a widespread phenomenon in taxa that are exposed to variable and dynamic environments. While some taxa may acclimate through phenotypic plasticity, or just modify their geographic distribution or behavior to avoid thermal stress [1,2], many others may evolve adaptive responses to local thermal regimes over generations via natural selection [3]. Such evolutionary adaptation to local thermal conditions has been verified across a wide variety of taxa [4,5,6,7,8], and is expected to play an important role in the survival of taxa with limited dispersals [3]. However, to date, the genetic basis of how various taxa acclimate these thermal stresses and adapt to environmental changes remains poorly understood. This is particularly true when it comes to the organisms in the marine realm, where analogous genetic information is yet to reach the same level as their terrestrial counterparts [9].

Mudflats are one of the most variable and dynamic habitats in the marine realm [10]. Taxa inhabiting this challenging environment may experience severe selection pressure from both abiotic and biotic conditions [11,12,13]. Particularly, mudflat species are constantly exposed to a range of environmental thermal stressors, including great variation in temperature from both temporal and spatial fluctuations [14,15]. As a consequence of this long-term selection, pronounced phenotypic plasticity and evolutionary adaptation may evolve [16], and thus provide a model system to investigate the genetic basis of adaptation to environmental thermal stresses. However, until now, both the prevalence and the exact molecular mechanisms underlying such a pattern of thermal adaptation remain largely unknown.

Fortunately, remarkable progress in molecular techniques such as RNA-seq provides the opportunity to investigate the transcriptional response to thermal stress and further identify molecular mechanisms and biological pathways involved in these thermal adaptations. The RNA-seq holds prospects for research of thermal adaptations, because acclimation and adaptation to thermal stress usually involve functionally tuning of a diverse variety of molecules, cell types, and organs to respond to thermal challenges [17,18]. During these processes, modulation of gene expression, the primary mechanism through which information encoded in the genome is converted into physiological phenotypes, is always involved [19]. Thus, identification of the key gene regulatory networks associated with thermal resistance would provide valuable information about the molecular mechanisms underlying the thermal adaptation [20]. A frequently used approach is to compare patterns of differentiation in gene expression between two closely related species with divergent thermal tolerance under the same thermal challenge [21,22]. In such a scenario, genes that affect the fitness of species under thermal challenge may also likely exhibit differential expression regulation between thermally tolerant and susceptible taxa [23]. Therefore, comparative studies of gene expression profiles among species with divergent thermal tolerance offer an opportunity to gain important insights into the mechanistic linkages between gene expression, biochemical pathways, and physiological function that underpin acclimation and adaptation to environmental stresses.

**Figure 1:**
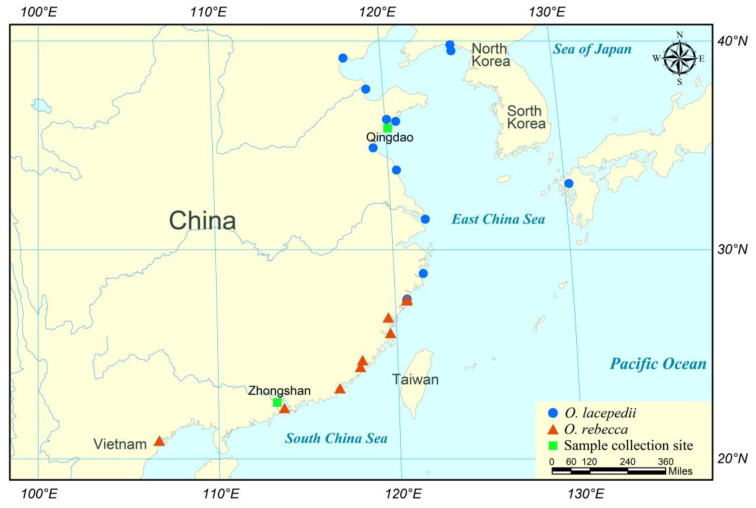
Distribution and sampling location of *O. lacepedii* and *O. rebecca*. The information on the natural distribution of two eel gobies was recompiled from Tang (2010) [24] with additional information from other authors: Lü (2023) [25]; Murdy (2003) [26]; Liu (2016) [27].

Here we assessed the gene expression divergence and thermal tolerance variation in two closely related mudflat inhabitants, *Odontamblyopus lacepedii* and *O. rebecca*, with contrasting latitude affinity, to investigate how gene expression is differentially regulated to enable thermal adaptation in tidal mudflat ectotherms. Both species are important components of the genus *Odontamblyopus* (Gobiidae: Amblyopinae) which are usually found in deep burrows of tidal mudflats, muddy bottoms of estuaries, as well as substrates from the adjacent shallow waters along the coast of China [24]. Though long considered as closely related sister species, divergent ecological preference has been recorded for the two species, with *O. lacepedii* endemic mostly to the North of the China Sea (primally from Liaoning to Zhejiang coastal waters) and *O. rebecca* primarily to the South China Sea (mainly from Zhejiang to Haiphong coastal waters), leaving a narrow contact zone located between them around the Taiwan strait regions (Figure 1) [24,25]. Such variation in latitude affinity may suggest divergent thermal tolerance and climate adaptation in these two eel gobies. Our previous analyses based on the whole genome re-sequencing also lent good support for their divergent thermal adaptation by revealing substantial signatures of selection on the DNA sequence level [25]. Despite these efforts, critical information is still lacking concerning exactly what level of thermal resistance diverges between each other, and how gene expression regulation is involved to underpin the divergent phenotype of thermal tolerance which potentially exists between the two taxa. The outcome of the research will not only provide new insights into how teleosts could cope with thermal stress, and their potential evolutionary strategies for adapting to their thermal environment, but also has good implications in sound management and conservation of their fishery resources in a scenario of global climate warming in the marine realm.

## 2. Materials and Methods

### 2.1. Sample Collection

Samples of *O. lacepedii* and *O. rebecca* were, respectively, collected from coastal waters of the north Yellow Sea in Qingdao of Shandong Province (120°19′ E, 36°16′ N), and the South China Sea in Zhongshan of Guangdong Province (113°36′ E, 22°30′ N) (Figure 1) using the ground cages in October 2022. For each species, a total of 100 individuals were collected and then immediately transported to the laboratory alive in plastic bags containing plentiful water and oxygen (one-third of water, and two-thirds of pure oxygen). After sample arrival, they were maintained at 23 °C in a recirculating system of 50 L aquaria filled with aerated seawater. An acclimation temperature of 23 °C was selected because it is probably appropriate for both species, considering that it falls into a cope of normal temperatures fluctuation (3.0–26.7 °C and 16.9–29.9 °C) (Table 1) in natural habitat for both species, and fish assemblage or spawning event occur when this ambient water temperature arrives (e.g., *O. rebecca* assembles in Zhongshan coastal waters when the temperature is 24.7 °C [28] and *O. lacepedii* spawns in Tianjin coastal water when the temperature is 21.5–25.5 °C [29]). The eel gobies were fed with small live shrimp *Penaeus vannamei* twice a day and acclimated for two weeks before experiments to allow the fish to recover from the adverse effects of transportation. After the acclimation, the fishes were starved for 24 h to eliminate the adverse impact of digestive activity on the experiment [30,31] before the assessment of thermal tolerance variation, as well as the gene expression divergence. All animal handling in the experiments as well as tissue sampling procedures afterward conform to all relevant ethical regulations provided by the Institutional Animal Care and Use Committee of Zhejiang Ocean University.

### 2.2. Determination of Cold Tolerance Capacity in the Two Eel Gobies

A parameter of critical thermal minimum (CT_min_) was used to compare the cold tolerance capacity between the two species of eel gobies. Here, CT_min_ was defined as the arithmetic mean of the collective thermal points at which the endpoint is reached according to Currie (1998) [32], which is commonly used as a proxy for cold tolerance in fish [33,34]. For the assessment of CT_min_ in each species, three groups of randomly selected individuals, with ten individuals for each group, were removed from the acclimation aquaria and introduced into three new testing aquaria. Water quality variables in the CT_min_ testing chamber were matched to those of the acclimation aquaria before the introduction of fish. Test water was continually mixed and aerated during trials by bubbling air through air stones. The water temperature in each testing aquaria was regulated by a Blue-M constant flow portable cooling coil during CT_min_ trials. During the experiment, water temperatures were decreased by activating the cooling element of one or more circulating thermoregulators. Water temperature was decreased at a constant rate of 0.3 °C min^−1^ during CT_min_ trials, which follow the CTM criterion proposed by Becker & Genoway (1979) [35]. Individuals were observed continuously until they reached the end-point, which is defined as the loss of equilibrium, evidenced by the inability to maintain dorsoventral orientation for at least 1 min [32]. The temperature at which each fish reached its end-point was recorded and used to calculate the CT_min_ and its standard deviation with the formula ${CT}_{min}=sum\left(T_{LOEn}\right)/n$, where $T_{LOEn}$ represents the temperature at which each individual reaches their end-point [36].

### 2.3. Transcriptomic Response to Cold Stress in the Two Eel Gobies

#### 2.3.1. Cold Stress Experiments and Tissue Dissection

Another batch of 60 fish individuals, with 30 individuals for each species, were randomly selected and used for the cold stress experiments. For each species, individuals were randomly divided into three groups with 10 individuals for each group. One group was used as control, which was maintained at a constant acclimation temperature of 23 °C. The other two groups were used as cold stress groups, which were maintained at 15 °C and 11 °C, respectively. The temperatures chosen for the cold stress experiments were based on our results of the CT_min_ assessment described above. To induce cold stress, groups of fish individuals were introduced into the challenging aquaria as already depicted. A cooling element of circulating thermoregulators was activated to decrease the water temperatures at a rate of 5 °C/h until the temperature reached the preset cold stress level, and then maintained constantly for 48 h during the experiment. No fish died during the cold stress. After the cold stress, three individuals in each group were randomly selected as replicates. The fish were then anesthetized using 80 mg/L concentration of tricaine methanesulfonate (M222), and the brain, gills, and liver tissues from each individual were rapidly removed after anesthesia, and immediately snap-frozen in liquid nitrogen until use.

#### 2.3.2. RNA Isolation, Library Preparation and Sequencing

Total RNA was isolated from each tissue using the standard TRIzol method (Ambion/Invitrogen, Waltham, MA, USA) [37]. The quality and concentration of the isolated RNA were qualified using 1.5% agarose gel electrophoresis and Agilent 2100 bioanalyzer (Agilent Technologies, Santa Clara, CA, USA). The isolated RNA was then used to construct the RNA-seq libraries. In total, fifty-four RNA-seq libraries (three tissues with three replicates per species at each temperature) were constructed using the NEBNext^®^Ultra™ RNA Library Prep Kit for Illumina^®^ (NEB, Ipswich, MA, USA) following the manufacturer’s instructions. Libraries were then sequenced on an Illumina NovaSeq 6000 platform (Illumina, San Diego, CA, USA) at Beijing Novogene company (Novogene, Beijing, China).

#### 2.3.3. Reads Mapping and Gene Expression Comparison

After quality control of the raw sequencing data using Trimmomatic (v. 0.36) [38] to remove the adaptors and low-quality reads, clean reads were aligned to our previously sequenced reference genome of *O. lacepedii* (GenBank: GCA_032888595.1) using HISAT2 (v. 2.0.5) [39] with default parameters. The fragments per kilobase of exon model per million mapped fragments (FPKM) values were calculated using featureCounts (v. 1.5.0-p3) [40] and custom Perl scripts to estimate gene expression levels. Differentially expressed transcripts between each pair of the samples were identified using the DESeq2 package [41], and differentially expressed genes (DEGs) were defined by a threshold of |log2(Foldchange)| ≥ 1 and *P.*adjust ≤ 0.05 [42]. In addition, to investigate the dynamic changes in gene expression across temperatures and species, the calculated DEGs were further extracted and clustered according to their dynamic expression profiles across all the samples with the parameter “cluster_rows=T” in the “pheatmap” package in the R software (v 4.3.1) [43]. In this package, the optimal number of clusters was determined based on the Bayesian information criterion (BIC) [44]. The Euclidean correlation dissimilarity matrix was used to perform the Hierarchical clustering of genes based on the complete linkage method. Heat maps depicting profiles of gene expression across temperatures and species were also plotted. To enable a straightforward and simplified illustration of the dynamic gene expression profiles, average expression values of the three replicates at each temperature for each species were applied to plot gene expression alterations for each cluster of genes. The identified DEGs were also used for GO and KEGG enrichment analysis via the R package clusterProfiler (v. 3.8.1) [45]. Significantly over-represented GO terms and KEGG pathways were identified with a *p* value ≤ 0.05.

### 2.4. Identification of Cold Responsive Modules in the Two Eel Gobies

A standard two-step procedure was applied to explore the cold-responsive modules and hub genes possibly associated with the cold tolerance in eel gobies according to Li (2023) [46]. Firstly, a weighted gene co-expression network was constructed to identify the cold-responsive modules using the R package WGCNA (v. 1.68) [47] following the package tutorials. To this end, all genes with the fragments per kilobase of exon model per million mapped fragments (FPKM) values less than 0.5 in more than 90% of the samples were removed to avoid noise from lowly expressed genes. The expression of the resultant 24,013 and 23,185 genes was then normalized by applying the varianceStabilizingTransformation function from the R package DESeq2 (v. 1.42.0) [41]. The adjacency matrix was estimated using a soft thresholding power of 5, which was recommended by command ‘sft$powerEstimate’ in the WGCNA package according to our data (the package will select the optimal soft thresholding power after balancing the scale independence and mean connectivity of the WGCNA by achieving fine mean connectivity while meeting a minimum scale-free topology fit index of 0.85 required by WGCNA analysis (Figure S1) [48] to ensure a scale-free network, according to Wang’s criteria [49]). The topological overlap matrix was calculated with minModuleSize set to 350, and cut-off heights were set to 0.99. A gene dendrogram was then used to identify modules using the cutreeDynamic function. Gene modules corresponding to the branch cut-off of the gene tree were labeled in different colors. The overrepresentation of tissue-specific DEGs between the cold stress and the control group was then calculated for each module by a hypergeometric test to identify the cold-responsive modules (CRMs). Secondly, the hub genes in each module were identified by characterizing the intramodular connectivity (K_within_), which represents a gene’s connection strength to other genes within the module, and the top 5% of genes with the highest K_within_ were defined as hub genes. The hub genes and their corresponding networks were visualized using Cytoscape (v. 3.9.1) [50].

## 3. Results

### 3.1. Variation of Cold Tolerance between the Two Eel Gobies

The two eel gobies exhibited different responses to cold stresses when exposed to the same chilling regime. The *O. lacepedii* reached the loss of equilibrium at 0.61 ± 1.23 °C, which is significantly lower than the *O. rebecca*, at 9.57 ± 1.19 °C (*t*-test, *p* value = 1.24 × 10^−25^) (Table 1).

### 3.2. Statistics of Transcriptome Sequences

A total of 367.79 Gb of raw RNA-seq reads were generated from three tissues of the brain, gills, and liver in *O. lacepedii* and *O. rebecca* exposed to different cold stresses using transcriptome sequencing strategy. After filtering the low quality data, the resultant clean reads were summarized in Table S1. A total of 30,818 protein-coding transcripts were obtained after mapping the clean reads to the reference genome of *O. lacepedii*. The mapping rates ranged from 77.73% to 85.50% (Table S1). The gene expression profile was thus calculated from the read counts of each successfully mapped gene and revealed a high correlation coefficient (>0.898) (Table S2) of gene expression and fine reproducibility among the biological replicates. All these 30,818 protein-coding transcripts were then included in subsequent analysis.

### 3.3. Transcriptomic Response to Cold Stress in Two Eel Gobies

**Figure 2:**
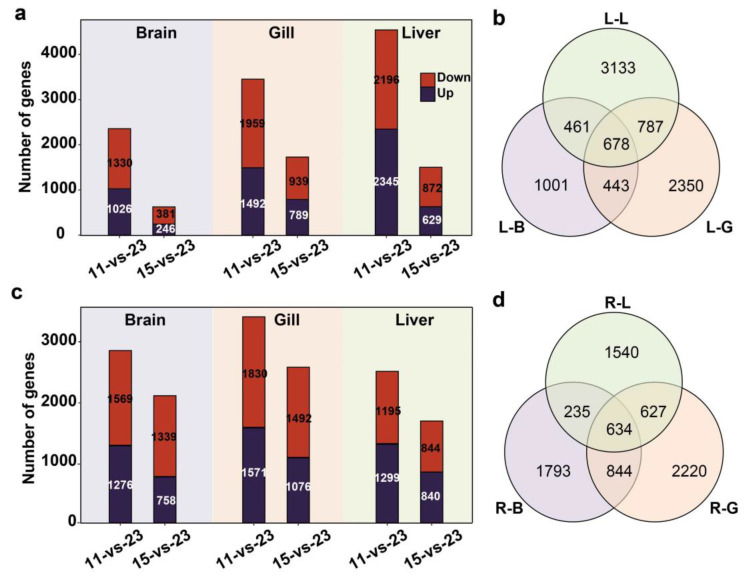
The number of differentially expressed genes (DEGs) in the brain, gills, and liver tissues of *O. lacepedii* and *O. rebecca* under different cold stress compared with the control groups (11-vs-23 °C, 15-vs-23 °C). (**a**) Number of DEGs in *O. lacepedii* under different cold stresses; (**b**) Venn diagram of the DEGs identified in the brain, gills, and liver tissues of *O. lacepedii* under cold stress (11 °C and 15 °C) compared to the control (23 °C); (**c**) Number of DEGs in *O. rebecca* under different cold stresses; (**d**) Venn diagram of the DEGs identified in the brain, gills, and liver tissues of *O. rebecca* under cold stress (11 °C and 15 °C) compared to the control (23 °C); The quantity of up and down-regulated genes were indicated by dark purple and red column, respectively, in the figure. L-B, L-G, and L-L represent the brain, gill, and liver tissues of *O. lacepedii;* R-B, R-G, and R-L represent the brain, gill, and liver tissue of *O. rebecca;* 11,15, 23 represent 11 °C, 15 °C, and 23 °C, respectively.

Analyses of the transcriptional changes in the brain, gills, and liver of both species exposed to cold stresses revealed a total of 8853 and 7893 cold-responsive DEGs in *O. lacepedii* and *O. rebecca*, respectively. Functional enrichment (GO) analysis revealed that the identified DEGs from the two eel gobies under cold stress were primarily enriched in multiple biological processes, such as nutrient metabolism, oxidation-reduction, cellular response to stimulus, circadian rhythm, DNA repair, covalent chromatin modification, apoptosis, immune response processes, cellular processes, protein folding, membrane organization, RNA processing, and energy metabolic processes (Tables S3–S14). The transcriptional changes in response to the cold stresses varied substantially across the temperatures, tissues, and species. When the effect of challenging temperatures was considered, more DEGs were up or down-regulated under rigid cold stress of 11 °C than the more moderate one of 15 °C in all three tissues examined in both species (Figure 2). Whereas, when the effect of species was partitioned, *O. lacepedii* usually had a lower transcriptomic response than their sister species of *O. rebecca* at moderate cold stress of 15 °C but climbed to almost the same level when challenged at more rigid cold stress of 11 °C (Figure 2). The liver seems to be the main responding organ in *O. lacepedii* since generally more DEGs were up or down-regulated in the liver under cold stress than the other two tissues (Figure 2), but gills seem to be the main target of cold response in *O. rebecca* for a much higher number of DEGs were activated in gills than the other two (Figure 2). Nonetheless, 193 common DEGs were also observed across all three tissues of the two species under cold stresses according to the Venn diagram constructed for illustrating the gene expression profiles across tissues (Figure S2, Table S15), which was primarily enriched in biological processes of carbohydrate biosynthesis (e.g., *MGAT2*, *NANS*, and *ISYNA1*), lipid metabolism (e.g., *PIGN*, *SC5D*, *ACOD*, *SDR42E1*, and *PLCG1*), and circadian rhythm process (e.g., *CIART*, *CIPC*, and *NFIL3*) (Table 2).

### 3.4. Distinct DEGs in the Two Eel Gobies under Cold Stresses

**Figure 3:**
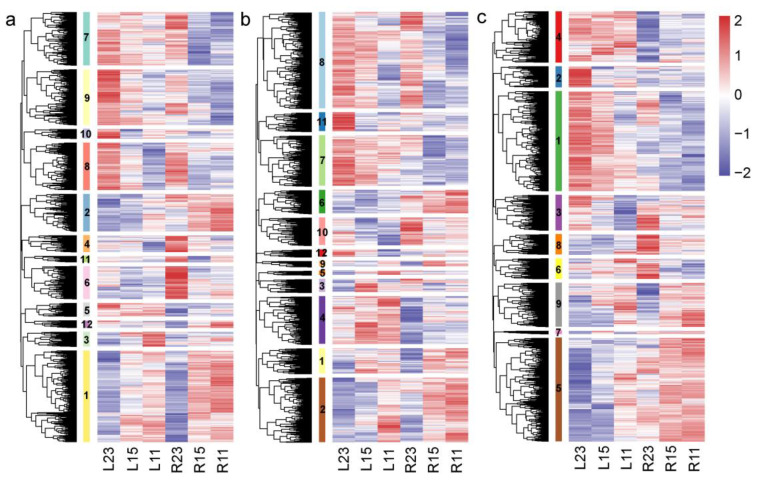
Heat map displaying the distinct gene expression patterns across various temperatures and species. Each row represents a unique gene, and each column represents a unique sample. The number represents different clusters. (**a**) DEGs identified in the brain tissues under cold stress are divided into 12 clusters based on their divergent expression profiles between the two species; (**b**) DEGs identified in the gill tissues under cold stress are divided into 12 clusters according to their divergent expression profiles between the two species; (**c**) DEGs identified in the liver tissues under cold stress are divided into nine clusters according to their divergent expression profiles between the two species. L-B, L-G, and L-L represent the brain, gill, and liver tissues of *O. lacepedii;* R-B, R-G, and R-L represent the brain, gill and liver tissue of *O. rebecca;* 11, 15, 23 represent 11 °C, 15 °C, and 23 °C, respectively.

**Figure 4:**
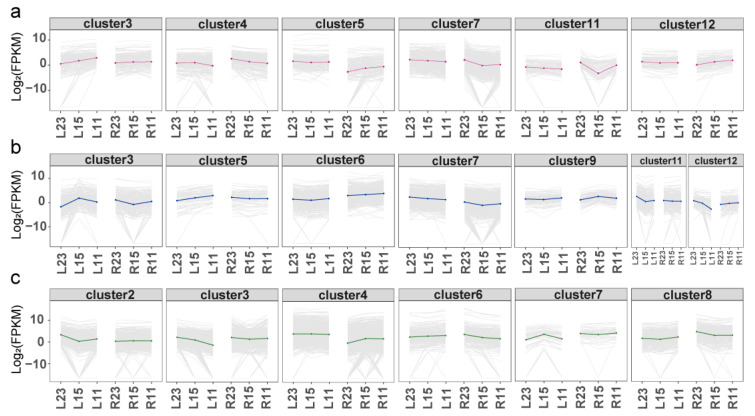
DEGs in three tissues that showing distinct expression profiles between the two species. (**a**) DEGs in the brain tissues that show distinct expression profiles between the two species. (**b**) DEGs in the gill tissues that show distinct expression profiles between the two species. (**c**) DEGs in the liver tissues that show distinct expression profiles between the two species. Dynamic gene expression profiles (gray lines) are depicted based on relative gene expression levels of individual genes at the three temperatures. Median values are used to generate a representative trend line in brain (red), gill (blue), and liver (green) tissues. Genes in these clusters show distinct expression profiles across species, both in terms of expression levels and responses to cold stress. In each graph, the labels on the x-axis from left to right designate samples from *O. lacepedii* (L) and *O. rebecca* (R) with 23, 15 and 11 represent control, cold stress at 15 °C, and 11 °C, respectively.

To better investigate the inter-specific distinct DEGs that are possibly related to the divergent cold tolerance capacity between the two eel gobies, a hierarchical clustering of genes based on the Euclidean correlation dissimilarity matrix was performed and the results revealed that multiple clusters exhibited distinct expression trends and responses to cold stress in three tissues between the two species. A total of 4943 DEGs were identified between the cold stress and control groups in the brain tissue of the two species. They were clustered into 12 groups (Figure 3a) based on the model-based clustering analysis implemented in the R package/mclust (Supplementary Table S16), among which, six clusters exhibited distinct expression trends and divergent responses to cold stress between the two species (clusters 3, 4, 5, 7, 11, and 12) (Figure 4a). The GO enrichment for DEGs in all six clusters revealed significant enrichment in biological processes associated with nutrient metabolism and energy production (GO:0019752; GO:0006082; GO:0043436; GO:0016051; GO:0005975; GO:0006520; GO:0006090; GO:0006096), DNA repair processes (GO:0006281; GO:0006974; GO:0006757; GO:0046031), and cellular component biogenesis (GO:0044085; GO:0030029; GO:0030036; GO:0071840) (Table S17). Similarly, in the gills, a total of 6863 DEGs detected under cold stress compared to the control groups in two species were clustered into 12 groups based on their expression patterns (Figure 3b). Among these clusters, seven displayed distinct expression trends between the two species (clusters 3, 5, 6, 7, 9, 11, and 12) (Figure 4b). Genes in this category were significantly enriched in nutrient metabolism and energy production (GO:0006508; GO:0006471; GO:0055114; GO:0009101), immune response (GO:0002376; GO:0006955), apoptosis processes (GO:0010941; GO:0042981; GO:0043067; GO:0006915; GO:0008219; GO:0012501), and DNA replication (GO:0006270; GO:0006261; GO:0006260), as well as in cell cycle process (GO:0022402; GO:0000278; GO:1903047) (Table S18). In the meantime, a total of 6881 DEGs were identified between cold stress and control groups in the liver tissue of the two species. They were clustered into nine groups based on their expression profiles (Figure 3c), among which, six clusters exhibited distinct expression trends and responses to cold stress between the two species (clusters 2, 3, 4, 6, 7, and 8) (Figure 4c). Genes in this category included several biological processes associated with nutrient metabolism and energy production (GO:0055114; GO:0005975; GO:0006082; GO:0043436 GO:0006508; GO:0030203; GO:0019752; GO:0010557); immune response (GO:0002376; GO:0006955) and DNA repair processes (GO:0006281; GO:0006974) (Table S19).

### 3.5. Cold Responsive Modules and Hub Genes Associated with Cold Tolerance

**Figure 5:**
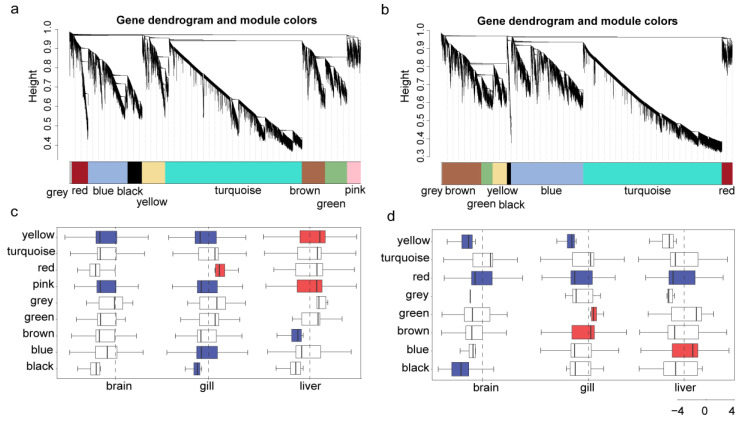
A weighted gene co-expression network analysis of genes expressed in three tissues of *O. lacepedii* and *O. rebecca*. (**a**) Clustering dendrogram of genes expressed in tissues of *O. lacepedii*. (**b**) Clustering dendrogram of genes expressed in tissues of *O. rebecca*. (**c**) The boxplot of nine modules identified in *O. lacepedii* based on the fold change of their expression levels. (**d**) The boxplot of eight modules identified in *O. rebecca* based on the fold change of their expression levels. The blue and red colors of the box represent down-regulated and up-regulated modules, respectively.

A total of 24,013 and 23,185 genes with the fragments per kilobase of exon model per million mapped fragments (FPKM) values ≥ 0.5 in more than 10% of the samples in *O. lacepedii*, and *O. rebecca* were selected for weighted gene co-expression network construction to identify the cold-responsive modules, respectively. The results showed that a total of nine modules with module sizes ranging from 220 (grey) to 11,290 (Figure 5a), and 8 modules with sizes ranging from 100 (grey) to 11,059 (Figure 5b) were assigned in *O. lacepedii*, and *O. rebecca.* Target modules associated with cold stress were further identified according to the hypergeometric test between DEGs and each module. The results revealed that six cold-responsive modules (CRMs) (*p* value < 0.05) were identified in both *O. lacepedii* and *O. rebecca*, according to the overrepresentation analysis. Among these, two, five, and three modules were identified as significantly correlated with cold stress in the brain, gills, and liver tissues in *O. lacepedii* (Figure 5c, Table S20), and three, four, and two modules were observed to significantly correlated with cold stress in the brain, gills, and liver tissues in *O. rebecca* (Figure 5d, Table S21).

**Figure 6:**
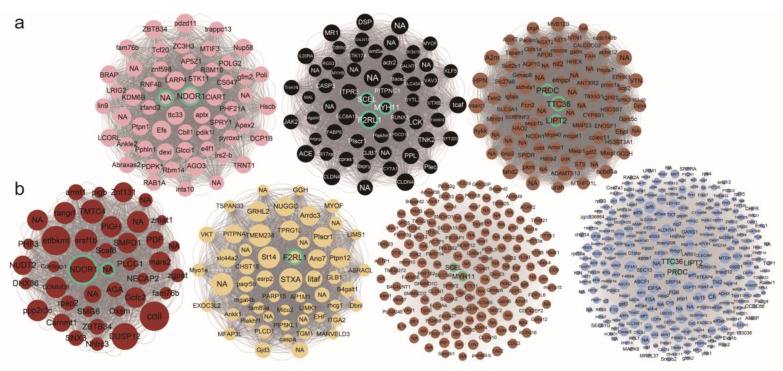
Network visualization of some hub genes (top 5% highest connectivity) shared between *O. lacepedii* and *O. rebecca* in cold-responsive modules. (**a**) Hub genes in *O. lacepedii*; (**b**) Hub genes in *O. rebecca*. Each colored node represents one hub gene in each module, with the name labeled. The size of the nodes represents the intramodular strength of connectivity, the higher the strength of the connectivity, the larger the size of the node. NA represents the gene without an annotation. The hub genes *F2RL1*, *LIPT2*, *NDOR1*, *TTC36*, *MYH11*, *SCEL*, and *PROC* were labeled with a green border color.

A further exploration of the gene connectivity using co-expression network analysis revealed a total of 150, 437, and 245 hub genes in the CRMs of the brain, gills, and liver tissue of *O. lacepedii*, respectively. Similarly in *O. rebecca*, 114, 296, and 326 hub genes were identified, respectively, in the CRMs of the corresponding tissues. These 1049 genes were highly connected to other genes in the corresponding modules. However, most of these cold-responsive hub genes are not shared between the two eel gobies of *O. lacepedii* and *O. rebecca* in all three tissues of the brain (98.46%), gills (92.68%), and liver (95.04%). Even in the 81 hub genes that were shared between these two sister species, eight of them involving those associated with energy production (*LIPT2*, *NDOR1)*, immunity response (*F2RL1*), and lipid metabolism (*MYH11*) still exhibited interspecific distinct expression trend and divergent responses to cold stress. (Figure 6).

## 4. Discussion

### 4.1. Species Divergency in Capacity of Cold Tolerance

Using the CT_min_ value as an indicator, the cold tolerance experiments revealed a much lower CT_min_ in *O. lacepedii* than *O. rebecca*, suggesting the northern cool-adapted species was more cold-tolerant than the southern warm-adapted one. This result is consistent with what could be expected from the ecological distribution of the two eel gobies along the coast of China. Such discrepancy in cold tolerance between the two species may be attributed to the contrasting latitude climate they experience, for the thermal tolerance of many organisms is usually proportional to the magnitude of variation in environmental temperature they combat [51]. Indeed, *O. lacepedii* in Qingdao coastal waters experiences northern temperate climates with the yearly average minimum–maximum surface water temperature of 3.0–26.7 °C (data from 2000 to 2022; https://downloads.psl.noaa.gov/Datasets/COBE/ (accessed on 1 September 2022)). In contrast, *O. rebecca* in Zhongshan coastal waters experiences a much more stable and warmer southern subtropical climate, with the yearly average minimum–maximum surface water temperature of 16.9–29.9 °C. Such contrasting differences in environmental thermal regimes may confer divergent thermal tolerance for mudflat inhabitants, such as the two eel gobies. Our results thereby indicate the possibilities of using latitudinal distributional information as one of the indicators for predicting the realistic patterns of species’ thermal tolerance in mudflat species, especially when their latitude distribution divergence is extreme, and thus may have positive implications for management and conservation of tidal fishery resources in the future.

### 4.2. Common Processes Involved in the Cold Stress Response

Since thermal stress has great biological impacts on organisms, the transcriptomic response is expected to be highly diverse across many genes in ectothermic species such as mudflat eel gobies. Our results were consistent with this expectation and found that numerous genes are differentially expressed in both species of eel gobies under cold stress. These DEGs were primarily enriched in multiple biological processes, such as nutrient metabolism, oxidation-reduction, cellular response to stimulus, circadian rhythm, DNA repair, covalent chromatin modification, apoptosis, immune response processes, cellular processes, protein folding, membrane organization, RNA processing, and energy metabolic processes (Tables S3–S14). Some DEGS include those genes (e.g., chaperones such as *HSP90*, *HSP10*) that are typically involved in cold stress response in a variety of animals [52,53]. These correspond well with previous studies, which demonstrate that all these biological processes are closely involved in cold tolerance when exposed to chilled waters in teleosts [54,55,56,57,58,59,60]. However, such transcriptomic response to cold stress was basically tissue specific both at the transcriptome or specific-gene expression level [61], as was also evident in other teleost fish when exposed to thermal stress [36,62]. The gill generally showed more DEGs up- or down-regulated under cold stress than the brain and liver (Figure 2) in *O. rebecca*. While the liver, instead, displayed more up- or down-regulated DEGs under cold stress than the other two tissues in *O. lacepedii*. The more DEGs regulated under cold stress is consistent with the expectation that gill and liver tissues are usually the main target organs of cold stress in fish and may play critical roles in their cold tolerance [63,64]. Nevertheless, a proportion of the DEGs exhibited conserved expression profiles across tissues and species, in terms of both expression levels and reaction to cold stress. These conserved genes probably represent components of the backbone of the stress response network in eel gobies that is essential for tolerance of thermal stress, which would make these genes interesting candidates to examine in other teleosts. Several genes (e.g., *ACOD*, and *ISYNA1*) associated with carbohydrate/lipid metabolism were found in this category and were significantly up-regulated in three tissues of both species during the cold stress. The carbohydrate/lipid metabolism pathway is the most frequently unveiled biological process that is closely related to cold resistance in a large variety of teleost species [3,55,60]. The parallel induction of these genes among tissues and species lent support for their roles in coping with cold stress in the eel gobies. Take *ACOD* as an example, this gene encodes Stearoyl-CoA desaturase that utilizes O_2_ and electrons from reduced cytochrome b5 to introduce the first double bond into saturated fatty acyl-CoA substrates and plays an important role in the regulation of cellular lipogenesis and membrane fluidity under temperature fluctuations [65]. In mice, ACOD deficiency will cause reduced adiposity and hence lower cold tolerance in a cold environment [66,67]. In aquatic oysters, cis- and trans-variations of stearoyl-CoA desaturase will cause large phenotypic plasticity for thermal adaptation in *Crassostrea gigas* and *C. angulate* [68]. In teleost fishes, activation of Stearoyl–CoA desaturase expression is usually parallel to the increased proportion of unsaturated fatty acids in cell membranes [69], hence the improved cold tolerance [70]. Therefore, we hypothesize that our observed up-regulation of *ACOD* expression may also be helpful in increasing the membrane fluidity, and hence the cold tolerance in eel gobies when exposed to cold stress possibly through lipogenesis stimulating processes.

Similarly, significant up-regulation in circadian rhythm-related genes such as transcriptional repressor (*CIART*), clock-interacting pacemaker (*CIPC*), and nuclear factor interleukin-3-regulated protein (*NFIL3*) were also found in in three tissues of both species during the cold stress. Circadian rhythm-related genes have been implicated in thermal resistance in a wide variety of teleost species such as Iberian chub (*Squalius carolitertii*) [62,71], sole (*Cynoglossus semilaevis*) [72], and tiger barb (*Puntius tetrazona*) [73]. Such correlation between circadian rhythm-related genes and thermal tolerance is hypothesized to be closely associated with the facilitating effects of circadian gene-dependent changes in cellular metabolism for cold resistance [74]. Indeed, So et al. (2022) first provided experimental evidence showing that protein encoded by *NFIL3* was found to participate in adipose tissue thermogenesis, in vitro. The knockdown of *NFIL3* disrupted the thermogenesis of systemic energy homeostasis in the adipocyte cell line of mice [75]. Thus, we postulated that the up-regulation of these circadian rhythm-related genes may also increase cold resistance in the eel goby by acting on the cellular metabolism processes such as energy homeostasis, as well. Further studies are needed to provide a solid linkage between the expression regulation of circadian rhythm-related genes and the phenotype of cold resistance in teleost fishes.

### 4.3. Distinct DEGs Corresponding with Divergent Thermal Adaptation

In addition to these common processes involved in the cold stress response, inter-specific distinct DEGs that are possibly related to the divergent cold tolerance capacity were also obvious between the two eel gobies. Our results of hierarchical clustering analysis revealed multiple clusters exhibiting species-distinct expression trends and responses to cold stress in all three tissues of the two species. Our weighted gene co-expression network construction and co-expression network analysis performed afterward also revealed numerous species-distinct cold-responsive hub genes that may be closely associated with divergent responses to temperature changes. Such interspecific distinctness in both categories and expression profiles of cold-responsive hub genes may arise from an adaptive alteration in the gene-expression-regulation network that may evolve under directional selection and confer the contrasted cold resistance capacity between the two eel gobies. This evolutionary force has been widely detected in other taxa of teleost species such as in *Oncorhynchus mykiss* [3] and *Lota lota* [76], where divergence in gene expression does not accord with genetic relatedness but is associated with phenotypic traits. Even in the hub genes that were shared between the two sister species, such interspecific variation of gene expression was also prevalent. A closer inspection of these shared hub genes exhibiting divergent expression between *O. lacepedii* and *O. rebecca* revealed that these genes were primarily associated with biological processes of nutrient metabolism, energy production, and immune response, which were typically involved in the cold stress adaptation in teleost species [55,77]. Among them, *MYH11* encodes smooth muscle-specific myosin heavy chain (SM-MHC) that is typically involved in the cytoskeletal and contractile function of smooth muscle cells (SMCs) [78]. However, in recent years, *MYH11* has also been found to be expressed in a variety of tissues and is closely involved in other cellular functions such as adipogenesis and thermogenesis [79,80] and plays an essential role in maintaining the body’s energy balance during cold adaptation. Our results found constant up-regulation of *MYH11* expression in the gill tissue of *O. rebecca* from 23 °C to 15 °C and 11 °C, implying that high-level adipogenesis and thermogenesis may have been activated to supply energy for counteracting the adverse effect of cold stress. In contrast, *MYH11* expression was largely down-regulated in the gill tissue of *O. lacepedii* from 23 °C to 15 °C, and was slightly increased from 15 °C to 11 °C. Such discrepancy in *MYH11* expression profile may imply that less adipogenesis and thermogenesis activity and less energy consumption was adopted to maintain the energy homeostasis in *O. lacepedii* than their sister species when exposed to the same cold stress regime, and thus may contribute, at least in part, to the higher cold tolerance in *O. lacepedii*. A similar discrepancy was also observed in the gene expression profile in the shared hub gene of *LIPT2* between the two eel gobies. *LIPT2* encodes the enzyme that catalyzes the transfer of endogenously produced octanoic acid from octanoyl-acyl-carrier-protein onto the lipoyl domains of lipoate-dependent enzymes, and is closely involved in the post-translational modification of key energy metabolism enzymes in animals [81]. Mutation or abnormal expression of *LIPT2* will usually cause a mitochondrial lipoylation defect [82] and mitochondrial membrane potential collapse [81], leading to severe defects in the mitochondrial energy metabolism. Our results also reveal constant up-regulation of *LIPT2* expression in the liver tissue of *O. rebecca* from 23 °C to 15 °C and 11 °C, implying that more energy was produced to meet the body’s energy requirement in *O. rebecca* under cold stress. Instead, in *O. lacepedii*, *LIPT2* expression was steadily down-regulated from 23 °C to 15 °C and 11 °C. Such ability to sufficiently reduce energy consumption by repressing the metabolic genes is believed to confer increased fitness when exposed to cold stress where energy gain is extremely limited [83]. Though the exact molecular mechanism underlying this upgraded metabolism in the *O. lacepedii* is unknown, such energy conservation strategies have also been frequently found in other taxa that show higher thermal tolerance than their lower tolerant counterparts [62,83]. Therefore, we hypothesize that such energy-saving strategies through repression of a small subset of metabolic genes and fine-tuning of associated metabolic processes probably represent a common physiological mechanism that confers enhanced cold resistance in teleost species when exposed to cold stress.

## 5. Conclusions

Our study demonstrated the strengths of comparing transcriptomes of closely related species to shed light on the underlying molecular mechanisms that have led to divergent thermal tolerance that was commonly found among teleost species. Using large-scale transcriptome data, we revealed that, despite the contrasting capacity in cold tolerance between the two eel gobies, there are still substantial DEGs that showed similar expression profiles across the species, which may indicate their core roles in cold stress response machinery in eel gobies. However, species-specific transcriptomic responses to cold stress were also observed, revealing that some genes associated with energy and nutrient metabolism, along with the immune response and apoptosis processes, may likely contribute to the enhanced cold tolerance of *O. lacepedii*. Among them, a subset of metabolic hub genes showing distinct down-regulation in *O. lacepedii* when exposed to cold stress highlighted the energy-saving strategies in the enhanced cold tolerance of eel gobies. These results may provide new information for understanding how teleosts could cope with cold stress and their potential evolutionary strategies for adapting to a changing climate in the marine realm. Such adaptative information of gene expression regulation among or within species is recommended to be taken into account in the identification of local adaptations and the development of sound conservation strategies of fishery resources in a scenario of global climate warming.

## Figures and Tables

**Table 1:** The CT_min_ values measured for *O. lacepedii* and *O. rebecca*.

Species	Sample Locations	Number	Body Mass (g)	CT_min_ (°C)
*O. lacepedii*	Qingdao, Shandong Province (120°19′ E, 36°16′ N; 3.0–26.7 °C)	30	76.96 ± 14.09	0.61 ± 1.23
*O. rebecca*	Zhongshan, Guangdong Province (113°36′ E, 22°30′ N; 16.9–29.9 °C)	30	68.34 ± 9.48	9.57 ± 1.19

Note: 3.0–26.7 °C and 16.9–29.9 °C are the yearly average minimum–maximum surface water temperatures in Qingdao and Zhongshan coastal waters, respectively. These data are downloaded from the website of Bio-ORACLE https://www.bio-oracle.org/downloads-to-email.php?sent=1&name=pointpy%40163.com) (accessed on 1 September 2022) (data from 2000 to 2022).

**Table 2:** GO Enrichment for common DEGs observed across all three tissues of the two species under cold stresses compared with the control group (*p* value < 0.05).

GO ID	Description	GeneRatio	BgRatio	*p* Value	Count
GO:0016051	carbohydrate biosynthetic process	3/48	14/6996	1.05 × 10^−4^	3
GO:0006629	lipid metabolic process	7/48	192/6996	2.98 × 10^−4^	7
GO:0008610	lipid biosynthetic process	4/48	59/6996	6.73 × 10^−4^	4
GO:0007623	circadian rhythm	3/48	10/6996	2.00 × 10^−3^	3
GO:0048511	rhythmic process	3/48	10/6996	2.00 × 10^−3^	3
GO:0048523	negative regulation of cellular process	3/48	69/6996	1.16 × 10^−2^	3
GO:0008654	phospholipid biosynthetic process	2/48	25/6996	1.25 × 10^−2^	2
GO:0048519	negative regulation of biological process	3/48	80/6996	1.72 × 10^−2^	3
GO:0005975	carbohydrate metabolic process	4/48	162/6996	2.45 × 10^−2^	4
GO:0006886	intracellular protein transport	3/48	107/6996	3.66 × 10^−2^	3
GO:0048522	positive regulation of cellular process	2/48	49/6996	4.42 × 10^−2^	2
GO:0048518	positive regulation of biological process	2/48	51/6996	4.75 × 10^−2^	2

Note: GeneRatio represents the ratio of the number of DEGs annotated in the GO term to the total number of DEGs. BgRatio represents the ratio of the number of background genes annotated in the GO term to the total number of background genes.

## Data Availability

The transcriptome datasets used in this study have been deposited in the NCBI Sequence Read Archive (SRA) BioProject PRJNA1002179.

## Supplementary Materials

The following supporting information can be downloaded at: https://www.mdpi.com/article/10.3390/cimb46010012/s1, Figure S1: Determination of optimum soft thresholding power; Figure S2: Venn diagram of the DEGs identified in the brain, gills, and liver tissues of *O. lacepedii* and *O. rebecca* under cold stresses; Table S1: Summary of clean RNA-seq reads generated from transcriptome sequencing of three tissues in two eel gobies; Table S2: The correlation coefficients between the biological replicates of eel gobies; Table S3: GO enrichment analysis of the DEGs identified in brain of *O. lacepedii* exposed to 15 °C compared with control group; Table S4: GO enrichment analysis of the DEGs identified in brain of *O. lacepedii* exposed to 11 °C compared with control group; Table S5: GO enrichment analysis of the DEGs identified in gill of O. lacepedii exposed to 15 °C compared with control group; Table S6: GO enrichment analysis of the DEGs identified in gill of *O. lacepedii* exposed to 11 °C compared with control group; Table S7: GO enrichment analysis of the DEGs identified in liver of *O. lacepedii* exposed to 15 °C compared with control group; Table S8: GO enrichment analysis of the DEGs identified in liver of *O. lacepedii* exposed to 11 °C compared with control group; Table S9: GO enrichment analysis of the DEGs identified in brain of *O. rebecca* exposed to 15 °C compared with control group; Table S10: GO enrichment analysis of the DEGs identified in brain of *O. rebecca* exposed to 11 °C compared with control group; Table S11: GO enrichment analysis of the DEGs identified in gill of *O. rebecca* exposed to 15 °C compared with control group; Table S12: GO enrichment analysis of the DEGs identified in gill of *O. rebecca* exposed to 11 °C compared with control group; Table S13: GO enrichment analysis of the DEGs identified in liver of *O. rebecca* exposed to 15 °C compared with control group; Table S14: GO enrichment analysis of the DEGs identified in liver of *O. rebecca* exposed to 11 °C compared with control group; Table S15: Common DEGs shared across all three tissues of the two eel gobies under cold stress compared with the control group; Table S16: Summary of model-based clustering analysis for DEGs identified in three tissues; Table S17: GO enrichment analysis of the DEGs exhibiting distinct expression profiles in the brain tissues between the two eel gobies when exposed to cold stress; Table S18: GO enrichment analysis of the DEGs exhibiting distinct expression profiles in the gill tissues between the two eel gobies when exposed to cold stress; Table S19: GO enrichment analysis of the DEGs exhibiting distinct expression profiles in the liver tissues between the two eel gobies when exposed to cold stress; Table S20: Overrepresentation analysis of differentially expressed genes in the modules of *O. lacepedii*; Table S21: Overrepresentation analysis of differentially expressed genes in the modules of *O. rebecca*.
