# Supplementary material for: Comparative Transcriptome Analyses Provide New Insights into the Evolution of Divergent Thermal Resistance in Two Eel Gobies
Source: Curr Issues Mol Biol. 2023 Dec 25;46(1):153–70. doi: 10.3390/cimb46010012 (PMC10813846; doi:10.3390/cimb46010012)
Supplement: Supplementary file 1 [file cimb-46-00012-s001.zip › Supply_Figures.pdf]

current issues in
molecular biology

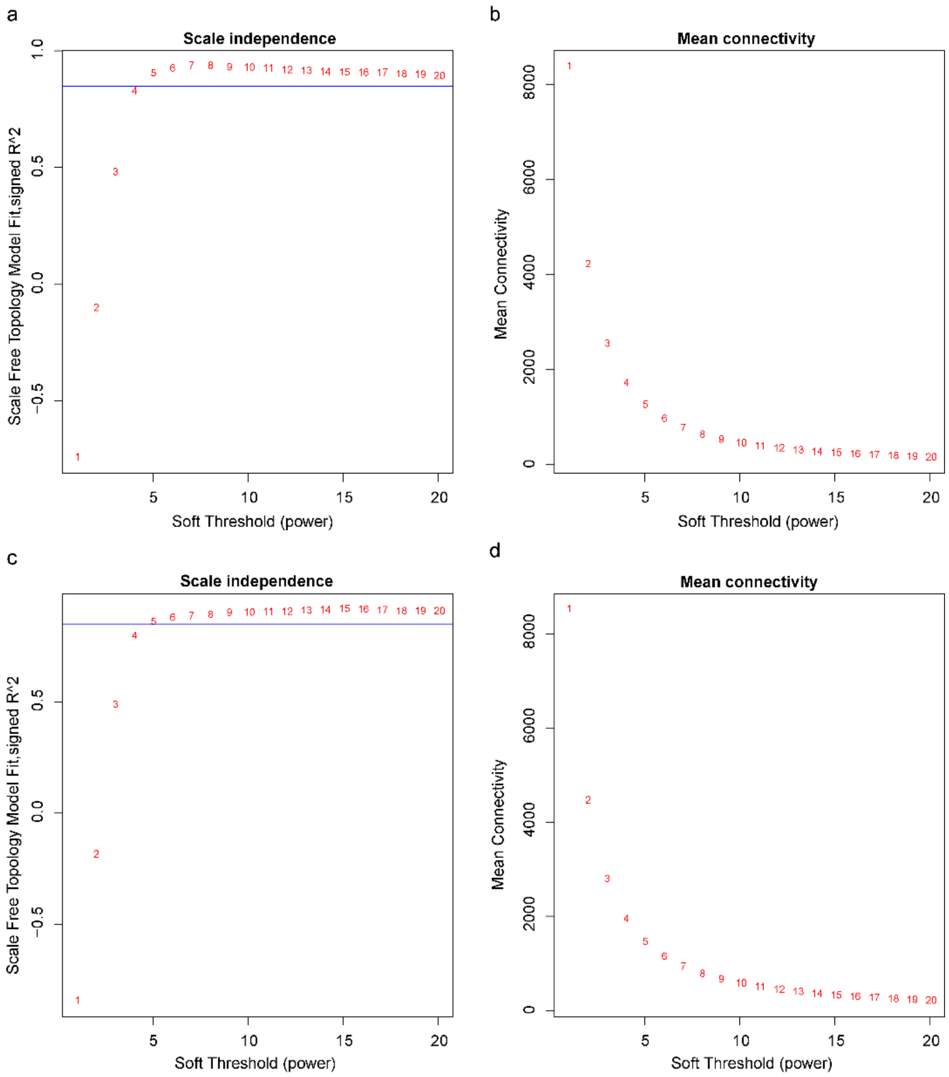

Figure S1. Determination of optimum soft thresholding power based on the analysis of network topology for various soft-thresholding powers of two eel gobies. **(a)** The scale-free topology fit index (y-axis) as a function of the soft thresholding powers (x-axis) in *O. lacepedii*; **(b)** The mean connectivity (y-axis) as a function of the soft thresholding powers (x-axis) in *O. lacepedii*; **(c)** The scale-free topology fit index (y-axis) as a function of the soft thresholding powers (x-axis) in *O. rebecca*; **(d)** The mean connectivity (y-axis) as a function of the soft thresholding powers (x-axis) in *O. rebecca*.

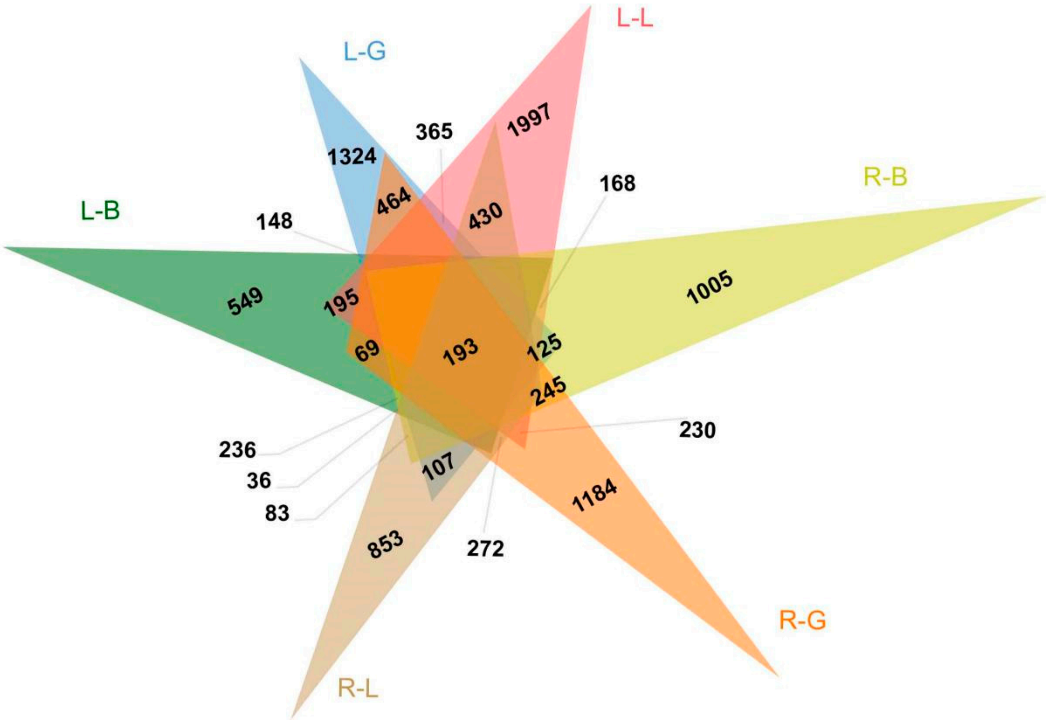

Figure S2. Venn diagram of the DEGs identified in the brain, gills, and liver tissues of *O. lacepedii* and *O. rebecca* under cold stresses. L-B, L-G, and L-L represent the brain, gill, and liver tissues of *O. lacepedii;* R-B, R-G, and R-L represent the brain, gill and liver tissue of *O. rebecca.*
